# Case of Compound Fracture of the Skull, with Symptoms of Compression—Operation

**Published:** 1856-12

**Authors:** Wm. Manson, John T. Richardson


ARTICLE II.—*Case of Compound Fracture of the Skull, with Symptoms of Compression—Operation.* By WM. MANSON, M.D. and JOHN T. RICHARDSON, M.D.

On the 10th of August, at 7 o'clock, A.M. we were called to

Michael Welsh, who the messenger said had been struck on the head with the pole of a hatchet. We arrived at the place in twenty-five minutes after the accident. Found the patient, (a native of Ireland, aged thirty-eight years, had enjoyed good health, but constitution somewhat impaired by intemperance,) with a bloody tumor of the scalp, over the anterior superior angle of the parietal bone of the left side. At the lower part of the tumor there was a wound, through which we could introduce the index-finger, and distinctly feel the sharp edge of the fractured bone.

There being all the symptoms of compression, we resolved to operate as soon as we could procure a trephining case, for which we were obliged to send twelve miles; and at $11\frac{1}{2}$ o'clock A.M. we commenced the operation by making T incision, which, owing to the extent of the fracture, we had to make into a crucial. After dissecting up the scalp, examining the extent of the fracture—which proved to be two inches and three-fourths in length, and one inch and three fourths in breadth, extending across the sagital suture and involving some of the opposite parietal bone, very irregular—we removed about half a dozen small pieces of comminuted bone, with a pair of dressing forceps, and there remained two large pieces. By sawing off a corner of solid bone, about three quarters of an inch, we took out one of the pieces, and then removing about an inch were enabled to take all out. But the internal table, extending in some places as much as half an inch farther than the external, we were obliged to saw around almost the entire fracture; Heys saw being the one used. The dura mater was ruptured in one place to the extent of half an inch. There was alarming hemorrhage from the rupture of a small artery, which was out of sight, but was controlled by sponges dipped in ice-water and applied to the wound.

The spiculæ of bone being removed by bone forceps, and the hemorrhage ceasing in about two hours from the commencement of the operation, we dressed the wound by placing back the flaps, using two or three sutures—keeping the edges of the flaps together mainly by adhesive straps.

The patient became rational during the operation, and it was with some difficulty that three men could keep him quiet. The skin had by this time began to grow warm; the breathing, from being stertorious, now became natural.

Left him in care of two trusty men; ordered him to be kept quiet, and applied cold water to his head. Gave two drops of croton oil, with orders to repeat it in half an hour, if it did not operate.

We visited the patient again at 6 P.M. Flighty at times; recognizes all who call in; feels no pain; pulse eighty-six, soft and regular; skin warm and a little moist; bowels not moved.

Ordered, hydrarg. S.M. gr.xxv. to be taken at 9 o'clock.

*Aug.* 11, 6 *P.M.*—Slept the most part of the night; bowels moved three times copiously during the night; pulse ninety, full and with considerable force; drank a little tea.

Ordered, potassœ antimonis tartras, in nauseating doses through the day.

Has fever; pulse 100, wiry; drinks copious draughts of water. Dressed the wound—healing by the first intention. The patient was raised to the sitting posture, and bled from the arm to approaching syncope, losing about twelve ounces of blood, which relieved the pain in the head. Was ordered to take the following:—

℞—Hydrarg. S.M. . . . gr.xij.
Pulv. Ipecac. . . . . gr.ij.
M. Divide into 6 powders;
One to be taken every four hours.

12*th*, *A.M.*—Passed a comfortable night; fever abated; bowels moved twice through the night; wound seems to be doing well.

6 *P.M.*—Much the same as in the morning. Entirely rational, but cannot command words to convey us ideas.

13*th*, 6 *A.M.*—Complains of pain about the wound; increased when he moved. Forgets sometimes what he is talking about. Pulse eighty, feeble. Gave chicken tea, brandy, and quinine.

14*th*, 6 *A.M.*—The wound has the appearance of sloughing where it was bruised with the hatchet. The sutures were all removed. There was considerable discharge from the wound of bloody matter.

6 *P.M.*—Has been restless through the day; pulse seventy-six, feeble. Removed a large portion of the flaps, with the forceps, that had sloughed. The incisions are again opening; applied argenti. nitras, ʒss. to the ℥j. of water. Continued the brandy and quinine.

15*th*, 7 *A.M.*—Rested well through the night; feels weak; pulse sixty-six, regular; seems to be acquiring some force; vomits after eating and drinking; wound indolent, and not healing.

Directed, brandy, ℥ss. and gr.ij. of quinine, four times in the day.

16*th.*—Much the same as yesterday; wound shows no inclination to heal. When the dressing was removed, some arterial blood escaped from under the skull. Applied the solution of nitrate of silver. Bowels being costive, was ordered a laxative. Continued the brandy and quinine.

17*th.*—The patient passed the night rather comfortable; pulse sixty-six; tongue furred; bowels moved; complains of some pain in the wound—not healing. Removed everything that would have any tendency to produce irritation.

Treatment: continued the tonics, with an anodyne at night.

18*th.*—Feels better than he has since he has been hurt; pulse seventy-two and regular; can sit up some; wound not sloughing any, begins to look healthy.

Treatment the same as yesterday.

19*th.*—Patient convalescing; appetite better; wound granulating, but the granulations are weak. Used stimulating dressing.

The patient continued to do well for about ten days, gradually gaining his strength, at which time he was attacked with inflammatory rheumatism in one knee, which was treated in the regular way—proving rather obstinate, greatly retarded the healing of the wound in the head.

His diet was farinaceous during the first weeks of his convalescence; but was subsequently left to his own taste.

He is now, at time of writing (Oct. 1,) entirely well, but has not yet resumed his occupation owing to weakness.

